# Sodium Transporters in Human Health and Disease

**DOI:** 10.3389/fphys.2020.588664

**Published:** 2021-02-25

**Authors:** Kenneth B. Gagnon, Eric Delpire

**Affiliations:** ^1^Department of Internal Medicine, UT Southwestern Medical Center, Dallas, TX, United States; ^2^Department of Anesthesiology, School of Medicine, Vanderbilt University, Nashville, TN, United States

**Keywords:** Na^+^/K^+^ pump, Na/Glucose transporter, Na^+^/H^+^ exchanger, Na^+^/Ca^2+^ exchanger, Na^+^ channels, Na-K-2Cl cotransporter, Na-Cl cotransporter, cell growth

## Abstract

Sodium (Na^+^) electrochemical gradients established by Na^+^/K^+^ ATPase activity drives the transport of ions, minerals, and sugars in both excitable and non-excitable cells. Na^+^-dependent transporters can move these solutes in the same direction (cotransport) or in opposite directions (exchanger) across both the apical and basolateral plasma membranes of polarized epithelia. In addition to maintaining physiological homeostasis of these solutes, increases and decreases in sodium may also initiate, directly or indirectly, signaling cascades that regulate a variety of intracellular post-translational events. In this review, we will describe how the Na^+^/K^+^ ATPase maintains a Na^+^ gradient utilized by multiple sodium-dependent transport mechanisms to regulate glucose uptake, excitatory neurotransmitters, calcium signaling, acid-base balance, salt-wasting disorders, fluid volume, and magnesium transport. We will discuss how several Na^+^-dependent cotransporters and Na^+^-dependent exchangers have significant roles in human health and disease. Finally, we will discuss how each of these Na^+^-dependent transport mechanisms have either been shown or have the potential to use Na^+^ in a secondary role as a signaling molecule.

## Introduction

During the 1962 America’s cup, President *John F. Kennedy* stated “*It is an interesting biological fact that all of us have, in our veins, the exact same percentage of salt in our blood that exists in the ocean, and, therefore, we have salt in our blood, in our sweat, in our tears. We are tied to the ocean. And when we go back to the sea, whether it is to sail or to watch it, we are going back from whence we came*” (from the *John F. Kennedy* Presidential Library and Museum). While the salt (sodium chloride or NaCl) concentration in our blood is four times lower than in the sea (140 mM vs 599 mM), the 35th President of the United States was right to contemplate the fact that we all carry a bit of the ocean with us (Delpire and Gagnon, 2018b). The concentration of sodium (Na^+^) in our blood, interstitial fluids, and extracellular spaces, if not as high as sea water, is however elevated (140 mM) compared to the concentration of Na^+^ inside our cells (10–30 mM), thereby establishing an inward concentration gradient across the membrane of the cell. This gradient is opposite to the outward potassium (K^+^) gradient created by low extracellular K^+^ (3.5–5 mM) and high intracellular K^+^ concentrations (130–140 mM). These opposing Na^+^ and K^+^ gradients, maintained by the energy-(ATP)-consuming Na^+^/K^+^ ATPase, generate a membrane potential across the plasma membrane, and create electrical signals in the form of action potentials that sustain cardiac muscle contraction and promote long distance neuronal communication. *John F. Kennedy* was also correct regarding salt being in our sweat and tears, as the production and secretion of these fluids involve the transport of Na^+^ and Cl^–^, and obligatory water. For more than 30 years, the study of monovalent Na^+^ ions have been focused on epithelial transcellular transport and propagation of action potentials in central and peripheral neurons of the nervous system. However, intracellular Na^+^ in epithelial cells may also initiate a cascade of signaling events that elicit a “mitogen-like” response (Stanton and Kaissling, 1989). Indeed, Na^+^ may regulate intracellular post-translational events such as glycosylation, phosphorylation, and even exocytosis (Stanton and Kaissling, 1989).

**FIGURE 1:**
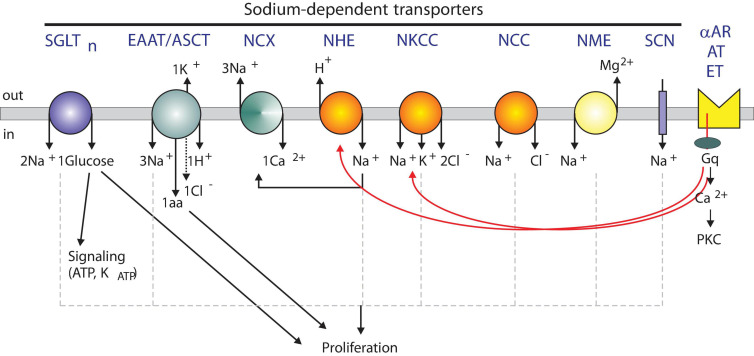
Model showing sodium-dependent transporters and possible signaling roles. Several Na^+^-dependent solute carriers with their individual stoichiometry of ions, sugars, and amino acids are depicted. SGLT, Na^+^-dependent glucose cotransporter; EAAT, Na^+^-dependent excitatory amino acid transporter; NCX, Na^+^-dependent Ca^2+^ exchanger; NHE, Na^+^-dependent hydrogen exchanger; NKCC, Na^+^-dependent potassium chloride cotransporter; NCC, Na^+^-dependent chloride cotransporter; NME, Na^+^-dependent magnesium exchanger; SCN, Sodium Channel. Transporters are regulated by alpha adrenergic (αAR), angiotensin II (AT), and endothelin (ET) receptors.

In this review, we will discuss the importance of Na^+^ transport, including how the Na^+^/K^+^ ATPase maintains a Na^+^ gradient utilized by multiple Na^+^-dependent transport mechanisms (see Figure 1) to regulate glucose uptake, excitatory neurotransmitters, calcium signaling, acid-base balance, renal salt reabsorption, and fluid and volume homeostasis, and discuss the role of each transporter/exchanger in human health and disease. Importantly, we will also cover evidence that the cation, in some well-establish cases, serves as a “signal” to affect metabolism, and promote cell division and cell growth. The Merriam-Webster dictionary defines a signal as “a detectable physical quantity or impulse by which messages or information can be transmitted” [“signal.” *Merriam-Webster.com.* 2020. https://www.merriam-webster.com (12 Sept, 2020)]. In the human body, signaling molecules, ranging from single ions to small proteins, are transmitted between different organ systems, within individual organs, amongst distinct cell populations, and within single cells. This intracellular and intercellular communication governs and coordinates basic cellular activity. In the spirit of this special series on the emerging role of monovalent ion transporters in intracellular signaling, we will address the limited but growing evidence that Na^+^ participates in signaling.

### Na^+^/K^+^-ATPase Creates the Primary Electrochemical Driving Force

The Na^+^/K^+^-ATPase is a plasma membrane “ion pump” that utilizes the energy produced from hydrolyzing the terminal phosphate bond of 1 ATP molecule to transport 3 Na^+^ out of the cell and 2 K^+^ into the cell. Cation transport against their concentration gradients requires cation binding sites with “locking gates” to occlude ion movement in the opposite, energetically ideal direction following conformational changes in the enzyme. Jens Christian Skou, awarded the Nobel Prize in Chemistry (1997) for the discovery of the Na^+^/K^+^-ATPase, outlined six requirements for this ‘ion pump’. It needed to: (i) be located in the cell membrane, (ii) have a higher intracellular affinity for Na^+^ versus K^+^, (iii) have a higher extracellular affinity for K^+^ versus Na^+^, (iv) have an enzyme capacity to hydrolyze ATP, (v) be able to hydrolyze ATP at a rate dependent on intracellular [Na^+^] and extracellular [K^+^], and (vi) be found in cells with active, linked transport of Na^+^ and K^+^ (Skou, 1957, Skou, 1965).

**FIGURE 2:**
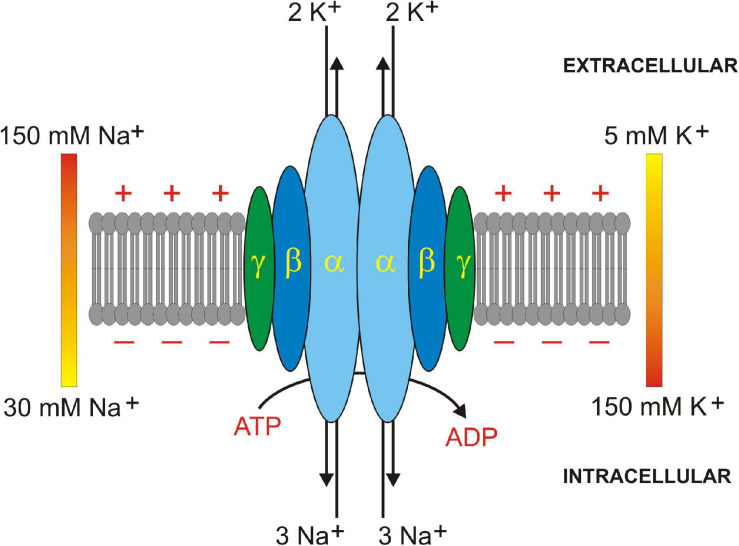
Na^+^/K^+^ ATPase subunit assembly in plasma membrane. Na^+^/K^+^ ATPase is a heterodimer of 1 alpha (α, light blue) and 1 beta (β, dark blue) subunit. A regulatory gamma (γ, dark green) subunit sometimes oligomerizes in some tissues. 3 Na^+^ and 2 K^+^ ions are translocated across the plasma membrane by hydrolysis of 1 ATP to 1 ADP molecule. Extracellular to intracellular ionic gradients for Na^+^ (150–30 mM) and K^+^ (5–150 mM) are shown. Positive and negative signs depict membrane potential created by Na^+^ and K^+^ ion translocation.

The Na^+^/K^+^-ATPase (see Figure 2) consists of an alpha (α) subunit (∼100 kDa) containing the catalytic site for ATP hydrolysis, a beta (β) subunit (∼45 kDa) critical for stability and trafficking to the membrane, and sometime, in specific tissues, a gamma (γ) subunit (∼10 kDa) that regulates Na^+^ and K^+^ affinity and cation uptake (Shinoda et al., 2009). There are four catalytic alpha subunits encoded on chromosomes 1p13.1 (ATPA1), 1q23.2 (ATPA2), 19q13.2 (ATPA3), and 1q23.2 (ATP1A4). There are three chaperone beta subunits encoded on chromosomes 1q24.2 (ATPB1), 17p13.1 (ATPB2), and 3q23 (ATPB3). Heterodimerization of one alpha subunit and one beta subunit allows for the possibility of 12 different Na^+^/K^+^-ATPase isozymes with tissue-specific distinct functional activities (Blanco and Mercer, 1998). There are also seven ‘FXYD’ gamma subunits that influence tissue-specific and isozyme-specific Na^+^/K^+^-ATPase activity and cellular function (Crambert and Geering, 2003). For an exceptional review on the physiological roles and tissue expression patterns of each of these Na^+^/K^+^-ATPase subunit isoforms, see (Clausen et al., 2017).

Alpha and beta subunits exhibit differential cation affinities and sensitivity to ouabain inhibition. Nearly every tissue expresses the α1 subunit, whereas, the α2 subunit is predominantly expressed in muscle, heart, brain, and adipocyte tissue. The α3 and α4 subunits are specifically expressed in nervous tissues and testes, respectively. Similarly, the β1 subunit is found in nearly every tissue, and accordingly, the α1β1 heterodimer is the most commonly expressed isozyme. The β2 subunit is found in muscle, pineal gland, and nervous tissue, whereas, the β3 subunit is expressed in the testis, retina, liver, and lung (Blanco and Mercer, 1998). Two recently published reviews from the Nissen group in Denmark characterize the structure and dynamics of these P-type ATPase ion pump isozymes (Dyla et al., 2019, 2020).

The ubiquitous expression of the various Na^+^/K^+^-ATPase isoforms in mammalian cells underlies its importance in maintaining Na^+^ and K^+^ electrochemical gradients across the plasma membrane. Multiple ion transporters and channels utilize these chemical gradients to drive ion, mineral, and sugar transport, and potentially downstream signaling pathways. As such, enzymatic dysfunction of the Na^+^/K^+^-ATPase, either through mutation, abnormal subunit dimerization, or abnormal membrane trafficking, has been linked to several human disorders. Multiple studies have associated Na^+^/K^+^-ATPase dysfunction with cancer initiation, growth, development, and metastasis (Babula et al., 2013). Cardiac glycosides have exhibited some anticancer effects via induction of apoptosis and autophagy, ROS production, and cell cycle arrest (Prassas and Diamandis, 2008). For more than 45 years, investigators have demonstrated a diabetic-induced decrease in Na^+^/K^+^-ATPase activity in the brain, heart, intestine, kidney, liver, and skeletal muscle (Vague et al., 2004).

Cardiac glycosides (e.g., digitalis) are routinely used to effectively treat patients experiencing heart failure. Studies have shown enzymatic inhibition leads to increased intracellular [Na^+^] (Erdmann et al., 1980; Grupp et al., 1985), resulting in lower driving forces for the Na^+^/Ca^2+^ exchanger, less Ca^2+^ extrusion from the cell, and ultimately more Ca^2+^ available to increase contractile force (Lee et al., 1985).

**FIGURE 3:**
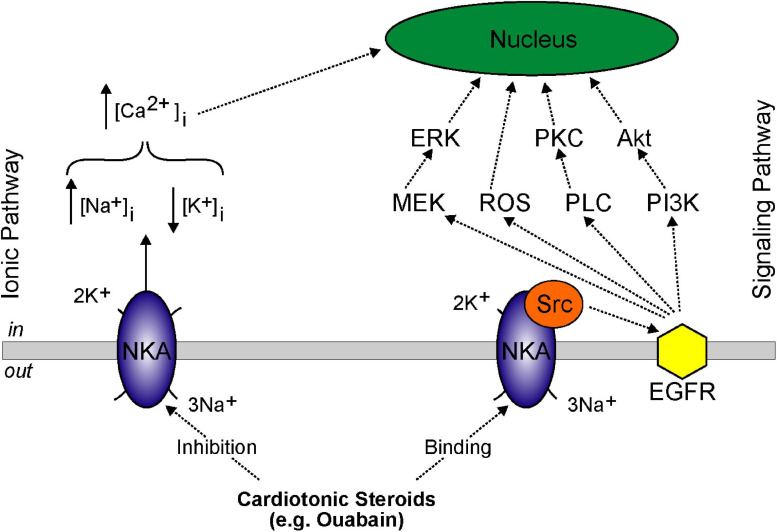
Model showing basolateral NKA ionic and signaling pathways. Ionic pathway involves: (i) high dose cardiotonic steroids inhibiting NKA activity, (ii) increased intracellular Na^+^ concentration, (iii) decreased intracellular K^+^ concentration, and (iv) increased intracellular Ca^2+^ concentration inducing genomic effects in the nucleus. Signaling pathway involves: (i) low dose cardiotonic steroid binding, (ii) release of Src, (iii) activation of EGFR, and (iv) signaling cascade activation of mitogen-activated protein kinase kinase (MEK/ERK), ROS generation, PLC/PKC activation, and PI3K/Akt activation.

In addition to being an “ion pump,” several studies over the past 20 years have shown the Na^+^/K^+^-ATPase to function as a receptor, signal transducer, and multi-protein scaffold (Liu et al., 2018). Sub-inhibitory concentrations of cardiotonic steroid binding has been shown to activate mitogen-activated protein kinase signal cascades, mitochondrial reactive oxygen species production, and the phospholipase C signaling pathway (see Figure 3) (Yuan et al., 2005). Binding of sub-inhibitory concentrations of ouabain have also been shown to induce activation of a signaling cascade leading to VSMC proliferation (Aydemir-Koksoy et al., 2001). As a protein scaffold, the Na^+^/K^+^-ATPase directly interacts and inhibits the non-receptor tyrosine kinase, Src. Following ouabain binding, the Src kinase domain releases from Na^+^/K^+^-ATPase and activates the EGFR forming a cardiotonic steroid receptor intracellular signaling pathway (Tian et al., 2006; Li et al., 2009). Other intracellular signaling proteins that have been shown to scaffold to Na^+^/K^+^-ATPase are ankyrin, cofilin, phospholipase Cγ, inositol triphosphate receptor, and phosphoinositide 3-kinase (Lee et al., 2001).

### Na^+^/Glucose Transporter (SGLT)

The first mammalian Na^+^/glucose cotransporter (SGLT) was cloned from rabbit intestine in 1987 (Hediger et al., 1987). Subsequent studies identified and cloned human SGLT1 and SGLT2 from kidney, thyroid gland, spinal cord, heart, muscle, and lung (Wright et al., 2011). Four isoforms (SGLT1, SGLT2, SGLT4, and SGLT5) of the SLC5 gene family transport glucose and/or fructose in a Na^+^-dependent fashion. Human SGLT1 is encoded on chromosome 22q13.1 and expressed in multiple tissues (small intestine, trachea, BBM late PT of kidney, heart, brain, testis, and prostate). Human SGLT2 is encoded on chromosome 16p12-p11 and initially was found only in the brush-border membrane of the early proximal tubule of the kidney. Interestingly, recent studies have identified expression of SGLT2 in brain, pancreatic, and prostrate tumors (Scafoglio et al., 2015). Human SGLT4 is encoded on chromosome 1p32 and expressed in the small intestine, kidney, liver, brain, and lung. Human SGLT5 is encoded on chromosome 17p11.2 and, to date, only found expressed in the kidney (Wright and Turk, 2004).

Functional studies have determined that SGLT2 is a low-affinity, high-capacity cotransporter with a one-to-one stoichiometry for Na^+^ and glucose. SGLT1 is a high-affinity, low-capacity cotransporter with a two-to-one stoichiometry for Na^+^ and glucose (Wright, 2001). The combination of internal and external Na^+^ and glucose concentration gradients and the cell membrane potential determine the direction and rate of Na^+^-dependent transport. As such, the transport cycle of SGLTs is completely reversible (Wright et al., 2017).

SGLT2, expressed in the S1 and S2 segments of the proximal tubule, reabsorbs 90% of the glomerular filtered glucose. SGLT1, expressed in the S3 segment of the proximal tubule, reabsorbs the remainder of glomerular filtered glucose resulting in negligible levels of glucose in the urine. Basolateral GLUT2 expression in all three segments completes transcellular transport of glucose across the tubule (Ghezzi et al., 2018). Several FDA-approved SGLT2 inhibitors (Farxiga, Invokana, and Jardiance) reduce blood glucose levels in patients with Type II diabetes (Gallo et al., 2015; Koepsell, 2017). However, patients with pharmacological inhibition or non-functional genetic mutations of SGLT2 have been found with only 50% glucosuria indicating that SGLT1 has a reserve capacity for glucose reabsorption in the proximal tubule S3 segment (Santer et al., 2003). Along with promoting glycosuria and improving glycemic control in Type II diabetics, pre-clinical and clinical studies have demonstrated multiple beneficial effects of SGLT2 inhibitors on cardiovascular and renal health, as well as treating certain cancers. Unfortunately, SGLT2 inhibitors can also lead to diabetic ketoacidosis, a metabolic condition where the body breaks down fat too fast and produces excess ketones causing the blood to become acidic (Perry and Shulman, 2020).

Autosomal recessive mutations in SGLT1 cause glucose-galactose malabsorption due to abnormal trafficking of the cotransporter in the intestine. Familial renal glucosuria is a result of autosomal recessive mutations that result in a premature stop mutation in SGLT2. Lastly, autosomal recessive mutations in GLUT2 result in Fanconi-Bickel syndrome (Santer et al., 2002; Santer and Calado, 2010; Wright et al., 2011). Although Na^+^ gradients established by Na^+^/K^+^ ATPase activity is essential to transcellular glucose transport in the kidney and intestine, SGLTs and GLUT2 cotransport of Na^+^ doesn’t meet the definition of a signaling molecule.

Na^+^-dependent transport of glucose is a key component of glucose blood homeostasis in all tissues (Berger and Zdzieblo, 2020). In pancreatic β-cells, transport of glucose through SGLT2 is an essential first step in a glucose-mediated signaling cascade that affects production of ATP and setting of an ATP/ADP ratio that ultimately controls the ATP-sensitive K^+^ channel (K_ATP_ channels), membrane depolarization, and release of insulin (Demirbilek et al., 2019). The SGLT2-mediated signaling uses glucose instead of Na^+^ as a mediator, but again – this is only possible because of the primary activity of the Na^+^/K^+^ ATPase that sets a highly favorable gradient for Na^+^-dependent glucose uptake.

### Na^+^-Dependent Amino Acid Transporters (EAATs, ASCT1/2)

There are seven members of the mammalian solute carrier family 1 (SLC1) consisting of five high-affinity excitatory amino acid (glutamate) transporters (EAATs) and two neutral amino acid (alanine, serine, and cysteine) transporters (ASCTs). Along with L-glutamate, the five EAATs also transport L-aspartic acid and are expressed in neurons, astrocytes, intestine, kidney, liver, heart, retina, and retinal cells (Kanai et al., 2013). As the primary excitatory neurotransmitter in the mammalian central nervous system, glutamate is involved in cellular migration, nervous system development, cognition, learning, memory, cellular differentiation, and neuronal death (Magi et al., 2019). Glutamate release into the synaptic cleft triggers AMPA glutamate receptors that are permeable to Na^+^, leading to membrane depolarization and action potential propagation. Na^+^-dependent glutamate transporters expressed in glia and neuronal postsynaptic boutons are essential to removing excitatory neurotransmitter from the synaptic cleft and preventing neurotoxicity. The two ASCTs mediate Na^+^-dependent exchange of the neutral amino acids L-alanine, L-serine, L-cysteine, L-threonine, L-glutamine, and L-asparagine. Both ASC transporters have widespread non-neuronal tissue expression including lung, skeletal muscle, intestine, kidney, testes, and adipose tissue (Kanai et al., 2013). As a result, dysfunction of these transporters are linked to metabolic reprogramming issues, renal pathologies, autophagy, tumor cell proliferation, and diabetes (Kandasamy et al., 2018).

### Na^+^/Ca^2+^ Exchanger (NCX)

Calcium (Ca^2+^) is an essential mineral necessary for life. Total human body Ca^2+^ ranges from 1,000 to 1,200 g with only 1% present in extracellular and intracellular spaces (i.e., 99% sequestered in the human skeleton) (Blaine et al., 2015). Despite the enormous disparity between free and sequestered Ca^2+^, free Ca^2+^ serves a vital role, including, but not limited to, hormone secretion, muscular contraction, blood coagulation, nerve impulse transmission, and intracellular adhesion (Hebert and Brown, 1996). Cytoplasmic concentrations of Ca^2+^ (∼100 nM) are maintained 20,000-fold lower than extracellular concentrations through plasma membrane Ca^2+^ ATPase-dependent (PMCA) and sarco(endo)plasmic reticulum Ca^2+^ ATPase-dependent (SERCA) mechanisms (Brini and Carafoli, 2009). Sudden and deliberate increases in cytoplasmic Ca^2+^ levels, either through internal release from ER/SR stores or external Ca^2+^ entry through plasma membrane ion channels, drives intracellular processes by exerting direct and indirect regulatory effects on a multitude of enzymes and proteins (Clapham, 2007). As a ubiquitous second messenger, Ca^2+^ mediates a wide-range of physiological events in nearly every cell of the human body. The speed, amplitude, spatial, and temporal patterns of intracellular [Ca^2+^] changes permits extensive versatility to Ca^2+^ signaling (Berridge et al., 2000).

The Na^+^/Ca^2+^ exchanger (NCX) directly couples the electrogenic transport of three Na^+^ and one Ca^2+^ ion across the cell membrane. Depending on ionic concentrations, net flux can either result in Ca^2+^ extrusion/Na^+^ entry (forward mode) or Ca^2+^ entry/Na^+^ extrusion (reverse mode) (Bers, 2002; Khananshvili, 2014). NCX1 was originally characterized from heart tissue and subsequently found to be highly expressed in brain, kidney, liver, pancreas, lung, placenta, and skeletal muscle (Kofuji et al., 1992). NCX2 and NCX3 protein expression appears to be unique to the brain and skeletal muscle (Li et al., 1994; Nicoll et al., 1996). Human SLC8A1 (NCX1) is encoded on chromosome 2p22.1 (Shieh et al., 1992), SLC8A2 (NCX2) is encoded on chromosome 19q13.3 (Li et al., 1994), and SLC8A3 (NCX3) is encoded on chromosome 14q24.1 (Nicoll et al., 1996). A fourth mammalian Na^+^/Ca^2+^ exchanger (NCLX) expressed in mitochondrial cristae of skeletal and cardiac muscle, pancreatic β-cells, and lymphocyte B-cells, and the brain plays a significant role in mitochondrial Ca^2+^ homeostasis (Palty et al., 2004, 2006, 2010; Lytton, 2007; Kim et al., 2012). Oxidative phosphorylation, synaptic transmission, cellular Ca^2+^ regulation, SR/ER Ca^2+^ content, hormonal secretion, and release of apoptotic factors are all fundamental processes dependent on mitochondrial Ca^2+^ homeostasis (Contreras et al., 2010; Drago et al., 2011; Mammucari et al., 2011).

NCX activity plays a pivotal role in Ca^2+^ balance during excitation (neuronal action potential) -contraction (muscle tissue) coupling. Overexpression/increased activity of cardiac NCX1 has been reported to contribute to (i) arrhythmias, (ii) heart failure, and (iii) myocardial ischemia-reperfusion injury (Egger and Niggli, 1999; Sipido et al., 2002; Pott et al., 2011). Several studies have implicated NCX1 upregulation in human vascular tissue to primary pulmonary hypertension (Nakasaki et al., 1993; Iwamoto et al., 2004; Zhang et al., 2010). In skeletal muscle, increases in Ca^2+^ lead to cross bridging of myosin-actin and contraction of muscle fibers (Baylor and Hollingworth, 2011). Similarly, control of myosin’s interaction with cytoskeletal actin by Ca^2+^/calmodulin regulates smooth muscle contraction/relaxation (Ledoux et al., 2006). Reduced expression of NCX1 and NCX3 results in intracellular Ca^2+^ excess and muscle degeneration (Kuyumcu-Martinez and Cooper, 2006). In neurons, cytosolic Ca^2+^ increases are important for electrical activity synchronization in the excitatory synapse (Franks and Sejnowski, 2002), neurogenesis (Rash et al., 2016; Toth et al., 2016), and synaptic plasticity (Hosseinian et al., 2020). Multiple studies suggest disruption of NCX expression/activity alters intracellular Na^+^ and Ca^2+^ homeostasis and may lead to several pathophysiological conditions (e.g., stroke and ischemia) in the brain (Pignataro et al., 2004; Jeffs et al., 2008; Molinaro et al., 2008). Increases in cytoplasmic [Ca^2+^] reduce and/or halt immune system cell motility (Gallo et al., 2006). NCX activity has been demonstrated to play a role in modulating Ca^2+^ signaling and cytokine production in human mast cells, macrophages, and monocytes (Aneiros et al., 2005; Staiano et al., 2009). Intracellular oscillations of Ca^2+^ in pancreatic β-cells is partially regulated by two NCX1 isoforms (NCX1.3 and NCX1.7) contributing to controlling [Ca^2+^] and release of insulin (Van Eylen et al., 1997; Ximenes et al., 2003). As a result, NCX1 expression/activity may have a possible involvement in the pathophysiology of type-1 diabetes and therefore may represent novel therapeutic targets (Herchuelz et al., 2007). NCX operating in reverse mode is a necessary step for vascular endothelial growth factor-induced ERK1/2 phosphorylation and angiogenesis in human endothelial cells, left ventricular fibrosis in cardiac fibroblasts, and nitric oxide-induced neuronal apoptosis in neuroblastoma cells (Andrikopoulos et al., 2011; Nashida et al., 2011; Kamimura et al., 2012).

Ca^2+^ surges released from internal stores during mammalian egg fertilization stimulates a signaling cascade resulting in the completion of meiosis and triggers specific mitotic events in the development of the embryo (Miyazaki et al., 1993; Swanson et al., 1997; Jones et al., 2000). Primary saliva production in parotid acinar cells is dependent on the coordination of intracellular Ca^2+^ signaling (Imbery et al., 2019). Finally, disruption of Ca^2+^ signaling can induce apoptosis through a complex interplay involving Ca^2+^ activated proteases, phospholipases, and endonucleases (Kass and Orrenius, 1999). The reversible role of Na^+^/Ca^2+^ exchange in multiple cell types and tissues underlies its importance in human health and disease. The question of whether Na^+^ or Ca^2+^ ions transported by NCXs participates as signaling molecules requires more investigation.

### Na^+^/H^+^ Exchanger (NHE)

The Na^+^/H^+^ exchanger belongs to the Slc9 gene family. Members SLC9A1–SLC9A5 (or NHE1-5) are targeted and expressed at the plasma membrane where they are activated by intracellular acidification and they function to exchange extracellular Na^+^ for intracellular protons (Fuster and Alexander, 2014). The first member, NHE1, was functionally identified by Pouysségur (Paris and Pouysségur, 1984). It is expressed in most mammalian cells. Intracellular acidification can occur in a variety of conditions, including ischemia (Karmazyn, 1996; Karmazyn et al., 2001) and cancer (Hulikova et al., 2013). The stoichiometry of exchange is strictly one for one and when the transmembrane concentration gradient for Na^+^ is balanced by an equivalent and opposite gradient for H^+^, the exchanger is at equilibrium and mediates no net ion flux (Grinstein and Rothstein, 1986).

While ischemic conditions lead to an intracellular acidification that is mitigated by activation of NHE1, the Na^+^/H^+^ exchanger paradoxically participates to cell injury (Karmazyn et al., 2001). The mechanism of injury involves activation of the Na^+^/Ca^2+^ exchanger due to Na^+^ accumulation in cells that have reduced Na^+^/K^+^ ATPase activity. Activation of the Na^+^/Ca^2+^ exchanger leads to Ca^2+^ overload and cell death (see previous section). Under normal conditions, the Na^+^ that enters the cell through the Na^+^/H^+^ exchanger is extruded by the Na^+^/K^+^ ATPase, but under ischemic conditions, ATP levels are low and consequently function of the Na^+^/K^+^ ATPase is reduced. In addition, some have speculated that the exchanger might also regulate the pH microenvironment of gap junction proteins and possibly of the sarcoplasmic reticulum Ca^2+^ release channel, i.e., components that likewise participate to the injury. Aside from intracellular acidification, the Na^+^/H^+^ exchanger is also activated by several hypertrophic factors such as α1-adrenergic stimulation, endothelin-1, and angiotensin II (Khandoudi et al., 1994; Matsui et al., 1995; Yokoyama et al., 1998). These receptors mediate activation of the heterotrimeric G protein, rise in calcium, and activation of PKC. While the mechanism of NHE1 activation is not fully understood, under these receptor agonist conditions, an increase in intracellular Na^+^ occurs, leading to an increase in cell volume, cell size, protein content, and ultimately tissue hypertrophy (Gu et al., 1998). Importantly, Na^+^/H^+^ exchanger inhibitors reduce this hypertrophic response (Kusumoto et al., 2001). In addition, absence of NHE1 expression provides some resistance to cardiac-ischemia reperfusion injury (Wang et al., 2003).

The Na^+^/H^+^ exchanger, like the Na-K-2Cl cotransporter, is also involved in cell volume regulation. When cells are exposed to hypertonic conditions, they first rapidly lose water and shrink to equilibrate inside/outside osmolarities. This shrinkage is often followed by a return to baseline volume through a process called regulatory volume increase (RVI). The Na^+^/H^+^ exchanger is a mechanism of RVI in lymphocytes (Grinstein et al., 1983), red blood cells (Cala, 1980; Parker, 1983) and other cells (Grinstein and Rothstein, 1986). An unexpected outcome came with the generation of the NHE1 knockout mouse. Mice lacking the exchanger exhibit diminished growth, ataxia, and epileptic-like seizures (Bell et al., 1999). The exchanger was shown to be critical in the response of neurons to acid load (Yao et al., 1999). Loss of either NHE1 or NHE2 also leads to reduced saliva secretion (Park et al., 2001). In colonic crypt epithelial cells, it is NHE2 that regulates intracellular pH and volume homeostasis (Bachmann et al., 2004).

There are also several forms of the Na^+^/H^+^ exchanger expressed in intracellular organelles. NHE6 is expressed in mitochondria (Numata et al., 1998), and endosomes (Brett et al., 2002), while members NHE7 and NHE9 are expressed in the Golgi. In these compartments, the exchanger regulates intra organelle pH to maximize their individual function. NHE9 is expressed in the inner ear and seems to prefer exchanging K^+^ instead of Na^+^ for protons (Hill et al., 2006).

### Na^+^-K^+^-2Cl^–^ Cotransporter (NKCC)

The Na-K-2Cl cotransporter (NKCC) mediates the coupled electroneutral movement of 1Na^+^, 1K^+^, and 2Cl^–^ ions across the plasma membrane. *SLC12A1*, located on the long (q) arm of human chromosome 15 (15q21.1) encodes NKCC2 – a cotransporter predominantly located in the thick ascending limb of Henle (hTAL), a portion of the kidney nephron involved in active trans-epithelial movement of Na^+^ (and Cl^–^), and secondarily of Ca^2+^ and Mg^2+^. Expressed at the apical membrane of the hTAL cells, NKCC2 facilitates the movement of Na^+^, K^+^, and Cl^–^ from the pro-urine into the cell, while the Na^+^/K^+^ pump on the opposite membrane moves Na^+^ into the interstitial space. This pathway contributes to 10–15% of the filtered Na^+^ being reabsorbed. By transporting K^+^ and Cl^–^, NKCC2 also actively participates in the creation of a driving force for the movement of Ca^2+^ and Mg^2+^ through the paracellular pathway. At the apical membrane, K^+^ ions entering the cell through NKCC2 leak back through K^+^ channels (ROMK) thereby creating an electropositive lumen. In addition, Cl^–^ ions co-transported with Na^+^ and K^+^ at the apical membrane, leak at the basolateral membrane through Cl^–^ channels (CLCK) creating an electronegative serosal side. The result of these two conductance is a trans-epithelial electrical potential that favors the movement of divalent cations through the narrow spaces existing between the epithelial cells and the tight junction proteins that form a significant barrier but are permeable to the divalent cations (Hou and Goodenough, 2010). Thus, each of the ions transported by this unique protein play a critical role in the overall function of this nephron segment. Loss-of-function mutations in NKCC2 results in a salt wasting disorder called Bartter syndrome type I (Simon et al., 1996), characterized by severe salt wasting, hypokalemic metabolic alkalosis and hypercalciuria (Fremont and Chan, 2012). The NKCC2 knockout mouse model dies before weaning due to failure to thrive and is non-viable unless treated with indomethacin from day 1 (Takahashi et al., 2000).

Note that while renal physiologists focus on Na^+^ reabsorption, they in the process mostly ignore Cl^–^, the accompanied anion. It is interesting that the opposite situation exists for NKCC1, the other isoform of the NKCC. NKCC1, encoded by *SLC12A2* a gene located on the long (q) arm of human chromosome 5 (5q23.3), is a widespread (but not ubiquitous) transporter and is mostly considered in terms of Cl^–^ transport. However, as a protein carrying Na^+^, K^+^, and Cl^–^ ions, NKCC1 participates in multiple functions ranging from Cl^–^ homeostasis (neurons, muscle) to trans-epithelial Na^+^ and Cl^–^ secretion (airway, fluid secreting glands, intestine), and even K^+^ secretion (inner ear). The role of NKCC1 in each of these functions will be discussed briefly.

Chloride (Cl^–^) is the major anion in the human body. Its concentration in the blood and extracellular fluid (95–110 mM) is relatively high. Inside cells, due to a large number of fixed anionic charges on macromolecules (Donnan effect) and a luminal-positive membrane potential generated by the leak of K^+^, the Cl^–^ concentration is generally much lower. It ranges from 5 to 10 mM in mature CNS neurons (Delpire and Staley, 2014) to 90–160 mM in red blood cells (Gunn et al., 1973). Most cells have an intracellular [Cl^–^] around 30–40 mM. NKCC1 actively participates in intracellular Cl^–^ homeostasis in both neurons and muscle cells. Indeed, NKCC1 is highly expressed in immature central neurons (Plotkin et al., 1997; Dzhala et al., 2005) as well as immature and mature peripheral neurons (Sung et al., 2000; Granados-Soto et al., 2005). By using the Na^+^ gradient generated by the Na^+^/K^+^ pump, NKCC1 can drive Cl^–^ uphill and accumulate the anion to levels that are far above its electrical potential equilibrium. In the young brain, because NKCC1 is expressed and KCC is not yet expressed, the Cl^–^ concentration is high, and opening of GABA_*A*_ receptors leads to giant depolarizing potentials (Ben-Ari et al., 1989; Ben-Ari, 2012). As the neurons mature, NKCC1 expression decreases and KCC2 (a neuronal-specific K-Cl cotransporter) expression increases, leading to much lower intracellular Cl^–^ concentrations that facilitate hyperpolarizing GABA responses and inhibition (Delpire, 2000; Delpire and Staley, 2014). Note that in the brain Cl^–^ ions might also participate in reciprocal signaling between neurons and astrocytes (Wilson and Mongin, 2019).

The situation is a quite different with peripheral neurons, as KCC2 is never expressed in the periphery and NKCC1 expression remains high in these cells throughout adulthood. As a consequence, the intracellular Cl^–^ concentration in sensory neurons is high (Alvarez-Leefmans et al., 1988), facilitating depolarizing GABA responses at the terminal of sensory fibers and presynaptic inhibition (Willis, 1999; Sung et al., 2000; Delpire and Austin, 2010).

NKCC1 is also expressed in skeletal (Gosmanov et al., 2003) and smooth muscle cells (Kaplan et al., 1996; Akar et al., 2001; Bulley and Jaggar, 2014) where it accumulates intracellular Cl^–^. High Cl^–^, in turn, allows for Cl^–^ channel-mediated membrane depolarization, thereby facilitating the opening of Ca^2+^ channels, the entry of Ca^2+^ into the cell, and muscle contraction. Thus, NKCC1 affects neurons and muscle cells function by regulating the level of intracellular Cl^–^. We will see below that these functions are affected in the NKCC1 knockout mouse and in the few individuals with mutations in the cotransporter.

NKCC1 plays an important role in Cl^–^ secreting epithelia, e.g., in shark rectal gland (Burger and Hess, 1960), mammalian salivary, sweat, lacrimal, pancreatic, gastric glands, but also in airway and intestinal epithelia (Grubb et al., 2001; McDaniel et al., 2005; Kidokoro et al., 2014; Delpire and Gagnon, 2016, 2018a). Located on the basolateral membrane, the cotransporter facilitates the trans-epithelial movement of Cl^–^ by bringing Cl^–^ into the cells for Cl^–^ channels (e.g., CFTR) which in turn transport it across the apical membrane. Thus, the combination of NKCC1 and CFTR function provides a pathway for transepithelial transport of Cl^–^. The Cl^–^ conductance at the apical surface is stimulated by cyclic adenosine monophosphate (cAMP), leading to a drop in intracellular Cl^–^. This, in turn, leads to the activation of With No lysine Kinases (WNK) which through SPAK activates NKCC1 to replenish intracellular Cl^–^ (Piala et al., 2014).

In the inner ear, NKCC1 fulfills a different function, namely participating in K^+^ secretion and the creation of a K^+^-rich endolymph. In the cochlea, the cotransporter is expressed in the stria vascularis (Crouch et al., 1997; Delpire et al., 1999), a multi-layer epithelium secreting K^+^. As it is the case for Cl^–^ secretion, NKCC1 on the basal side of stria vascularis epithelial cells replenish K^+^ as it is transported in the endolymphatic cavity by K^+^ channels formed by both KCNQ1 + KCNE1 subunits (Hibino et al., 2004; Rivas and Francis, 2005).

Importance of NKCC1 as a widespread protein was demonstrated with the development of knockout mouse models (Delpire et al., 1999; Flagella et al., 1999). NKCC1 knockout mice are severely deaf and suffer from inner ear balance deficit (Delpire et al., 1999; Flagella et al., 1999), have low blood pressure and decreased vascular tone (Meyer et al., 2002). They have deficits in saliva production (Evans et al., 2000), intestinal transit (Wouters et al., 2006; Zhu et al., 2016), intestinal hydration (Flagella et al., 1999; Koumangoye et al., 2020), deficit in goblet cell mucus release (Koumangoye et al., 2020). They also have alterations in airway ion transport (Grubb et al., 2001), display a pain perception phenotype (Sung et al., 2000; Laird et al., 2004), and the males are sterile (Pace et al., 2000).

Interestingly, to date, there are only three individuals with complete loss of function in NKCC1 (Macnamara et al., 2019; Stodberg et al., 2020). The case of a 5-year old boy who inherited, through uniparental disomy, two copies of chromosome 5 from his father (an asymptomatic carrier) was described. The boy suffers from hearing loss, developmental delay, intellectual disability, as well as deficits in sweat production, saliva production, lung and intestinal fluid production (Macnamara et al., 2019). Then there is the case of a compound heterozygous 8-year old girl having inherited from her mother a single base pair mutation of a splicing donor site leading to the skipping of exon 13, and from her father a 1 base deletion in exon 8 leading to an open reading frameshift. She suffers from severe neurodevelopmental disorder, hearing impairment, gastrointestinal problems, and deficit in sweat, tear, and saliva production (Stodberg et al., 2020). Note that this proband had an older sister that died 22 days after birth of cardiac arrest during assisted ventilation. Because her fibroblasts were collected, genetic analysis revealed that she carried both her parent’s mutations. Evidence is now mounting that *de novo* (single allele) mutations lead to neurodevelopmental deficits and cochlea-vestibular defects (McNeill et al., 2020; Mutai et al., 2020). Another patient with NKCC1 mutation deserves some attention in the context of this review article. A 17-year-old girl in California carries a *de novo* mutation in a single allele of exon 22 of *SLC12A2*, leading to premature termination of the open reading frame and truncation of the carboxyl-terminal tail of the protein (Delpire et al., 2016). For the longest time, her physicians and parents thought that she was suffering from some type of mitochondrial disease. As a baby, she was difficult to wake-up and otherwise would be in constant need of energy, as if her cells were in constant state of starvation. A muscle biopsy demonstrated increased mitochondrial DNA content, a sign of possible mitochondrial dysfunction (Delpire et al., 2016). From this description, it seems clear that she has a metabolic phenotype. Part of the issue might have been poor intestinal function, limiting the amount of nutrients absorbed from her diet. In fact, her treatment regiment included additional nutrition through a gastric tube, followed by a gastrostomy-jejunostomy tube, followed by a complete intravenous (parenteral) route. We were successful in creating a mouse model that recapitulated the patient mutation (Koumangoye et al., 2018). The mouse exhibited mis-trafficking of the cotransporter to the apical membrane of epithelial cells, decreased intestinal function due to deficit in luminal hydration, deficit in release and expansion of mucus layers (Koumangoye et al., 2020). As surprising, was our demonstration that cells from the mouse had increased mitochondrial respiration, and signs of starvation (Omer et al., 2020).

**FIGURE 4:**
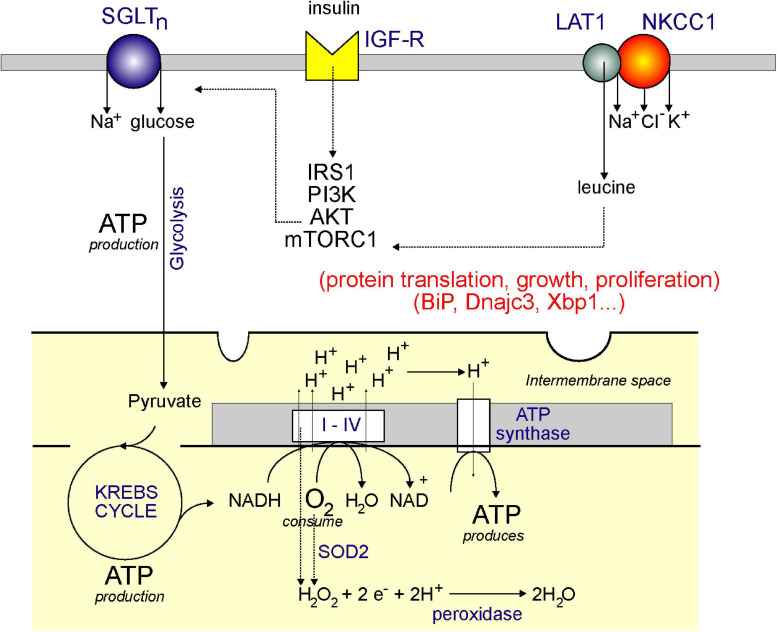
Model showing plasma membrane NKCC1 and mitochondrial respiration: a link through leucine? Aerobic respiration utilizes the KREBS cycle and the electron transport chain. Through the consumption of oxygen (O_2_) and NADH (produced by the KREBS/TCA cycle), complexes I–IV produces a proton (H^+^) gradient in the inner membrane of the mitochondria, which is then used by ATP synthase to produce ATP. The KREBS or TCA cycle is powered by pyruvate which comes from glucose through the process of glycolysis. The glucose comes from uptake at the plasma membrane through a Na^+^-driven glucose transporter. Glucose transport is facilitated by activation of the IGF-R receptor by insulin and stimulation of the IRS1/PI3K/AKT pathway. AKT also stimulates mTORC1, which stimulates protein translation, thereby facilitating cell growth and proliferation. mTORC1 is also stimulated by leucine which enters cells through the LAT1 transporter – which requires interaction with NKCC1. Note that mitochondrial respiration also produces hydrogen peroxide (through complexes I–IV and through SOD2), which can be converted to water by peroxidases.

**FIGURE 5:**
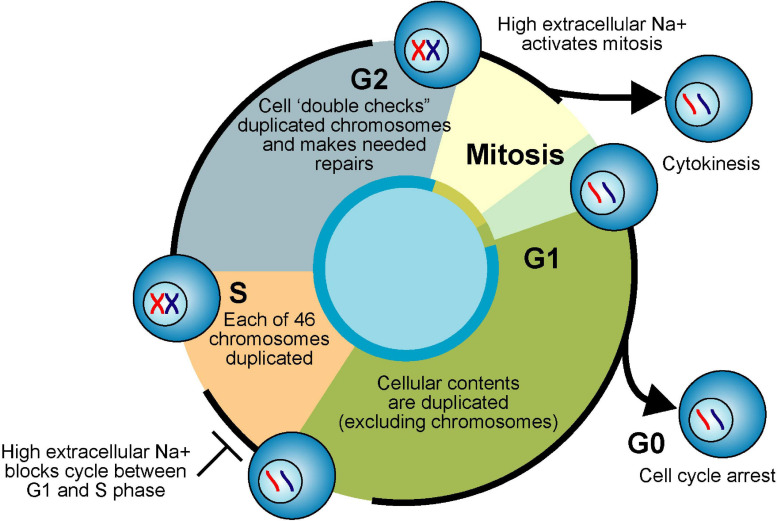
Cell cycle and Na^+^. Actively proliferating eukaryote cells pass through four phases of the cell cycle. Cellular contents (excluding chromosomes) are duplicated in first gap phase (G1). Each chromosome is duplicated in synthesis phase (S). Duplicated chromosomes are “error checked” during second gap phase (G2). G1, S, and G2 are collectively called interphase. Equal division of duplicated nuclear material occurs in mitosis phase (M). Division of parent cell into two daughter cells (cytokinesis) completes the cell cycle. If nutrients are limiting or the cells are fully differentiated due to internal genetic programming, cells exit interphase and enter a “resting phase” (G0).

Is NKCC1 involved in metabolism? A recent paper provides compelling evidence that NKCC1 is indeed involved in cellular metabolism (Demian et al., 2019). The authors demonstrated that NKCC1 function provides a break to the uptake of leucine through the LAT1 transporter and that reduction in NKCC1 function therefore facilitated leucine uptake leading to mTORC1 activation (Figure 4). This was shown through ShRNA-mediated downregulation of transporter expression, as well as through the use of the cotransporter-specific inhibitor, bumetanide. Activation of mTORC1 was blocked by PI3K and Akt inhibitors, as well as the insulin receptor inhibitor, indicating that signal leading to mTORC1 activation was mediated by both the receptor and the two kinases. Thus, NKCC1 activity affects mTORC1 independently through amino acid uptake and through the insulin receptor. Activation of mTORC1 by amino acid uptake ultimately led to the translocation of the complex to the lysosomal membrane. Inhibition of NKCC1 also led to enhanced cell proliferation and a redistribution of cells within the cell cycle. More cells were in the S phase, whereas fewer cells were in the G1 phase (see Figure 5).

Increased activation of the nutrient/energy sensor, mTORC1 is consistent with the increased need for mitochondrial respiration that we observed in cells isolated from the NKCC1-DFX patient and in cells from mice expressing the same mutant transporter (Omer et al., 2020). If NKCC1 functions as a break to the amino acid transporter LAT1 and to the insulin receptor, in macrophages, it also provides a break to the function of phagocytosis (Perry et al., 2019). In this case, however, it seems that intracellular Cl^–^ is a key component, as the process is slowed down by inactivation of KCC1 (SLC12A4) (Perry et al., 2019).

In the kidney, Cl^–^ plays an important role in the regulation of glomerular filtration. When filtration increases, the amount of Cl^–^ that is filtered increases and when the anion flows at the macula densa, it is “sensed” by a mechanism that involves NKCC2 on the apical membrane of macula densa cells which uptake NaCl and swell, thereby releasing ATP. The release of ATP activates A1 receptor on the afferent arteriole leading to contraction. Cl^–^ is also affecting the synthesis and secretion of renin through NKCC1 (Castrop et al., 2005), a cotransporter highly expressed in the renin-containing cells of the afferent arteriole (Kaplan et al., 1996). Under normal conditions, NKCC1 function keeps Cl^–^ high in renin cells, thereby tonically suppressing renin release and secretion. A drop in urinary Cl^–^ through NKCC2 will translate into a drop in interstitial Cl^–^ which then translates through NKCC1 into a decrease in intracellular Cl^–^, leading to increased renin secretion. Accordingly, in NKCC1 knockout mice, the basal levels of renin mRNA were elevated compared to wild-type mice (Castrop et al., 2005).

### Na-Cl Cotransporter (NCC)

The distal convoluted tubule (DCT) of the kidney reabsorbs a small, but critical, portion of the filtered body Na^+^ prior to being lost in the urine (Moes et al., 2014). Na^+^ reabsorption in the DCT is highly regulated and affects blood volume and blood pressure. It occurs mainly through the combined actions of the apical Na-Cl cotransporter (NCC) and the basolateral Na^+^/K^+^ ATPase (Gamba et al., 1994; Plotkin et al., 1996). Thiazide diuretics, for instance, which inhibit NCC function, are widely used in the clinic to treat volume expansion disorders such as congestive heart failure, kidney disease (Sica, 2003; Listed, 2014). Compared to other nephron segments, the DCT segment is relatively short. Its size, however, varies widely depending on the activity of NCC. To illustrate the tight relationship that exists between NCC function and morphology of the DCT, we will discuss analyses done in mice with manipulation of the Ste20p-related Proline-Alanine rich Kinase (SPAK, also known as STK39). SPAK is the major terminal kinase in the DCT that binds, phosphorylates, and activate the cotransporter (Vitari et al., 2005; Pacheco-Alvarez et al., 2006). Consistently, mice lacking SPAK display a Gitelman-like phenotype (similar to patients with loss-of-function mutations in NCC): being highly sensitive to dietary salt restriction and displaying prolonged negative sodium balance and hypotension (Grimm et al., 2012). As quantified by morphometric analysis using parvalbumin as a marker for DCT1, Grimm and colleagues showed a significantly smaller DCT1 in SPAK knockout mice (Grimm et al., 2012). In accordance with these observations, abundance of NCC and parvalbumin was significantly decreased, whereas abundance of calbindin (DCT2) and NKCC2 (TAL) was unchanged. The authors stated “*these observations provide unambiguous evidence that the mass of the DCT, specifically the DCT1, is reduced in SPAK null mice*”. Similar observations and conclusions were made in an independent study that used the same SPAK knockout mouse model (McCormick et al., 2011). Note that the same phenotype is observed in thiazide diuretic treated rats (Loffing et al., 1996) and in global NCC and parvalbumin-tissue specific NCC knockout mice (Loffing et al., 2004; Belge et al., 2007). In each case, NCC function is reduced or eliminated and the intracellular Na^+^ concentration is decreased. Importantly, the reverse situation also provides compelling evidence for the relationship between NCC, Na^+^, and tissue mass. Indeed, mice expressing a constitutively active SPAK display high blood pressure with hyperkalemia and metabolic acidosis without changes in plasma aldosterone or creatinine clearance. In this mouse model, NCC abundance and phosphorylation was enhanced and hypertrophy of the DCT was observed (Grimm et al., 2017). Both parvalbumin-positive DCT1 tubule length and cross-sectional area were expanded in these mice, compared to controls, while the mass of the more distal CNT (connecting tubule) significantly decreased.

The Cl^–^ regulation of WNK has been best studied in the DCT which constitutes a sensor for plasma K^+^ (Hoorn et al., 2020). A change in plasma [K^+^], through Kir4.1/1.1 channels, will lead to parallel changes in intracellular K^+^ and Cl^–^ concentrations in the DCT (Su et al., 2019). A decrease in plasma K^+^ results in a decrease in Cl^–^ in DCT leading to stimulation of the WNK4/SPAK cascade and to the activation of NCC and Na^+^ reabsorption. Conversely, an increase in plasma K^+^ leads to an increase in intracellular Cl^–^, inhibition of WNK4/SPAK and NCC, and to decreased Na^+^ reabsorption (Terker et al., 2015; Ellison et al., 2016).

**FIGURE 6:**
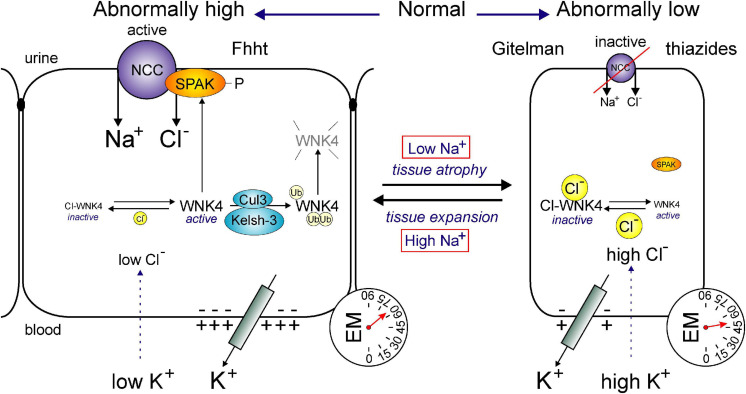
Hypertrophy or atrophy of DCT segment as a function of Na^+^ transport. Function of the NCC affects the size of the DCT segment. In conditions of abnormally high transport (left), such as Familial hyperkalemia and hypertension (Fhht, or Gordon syndrome) or low plasma K^+^, the DCT segment hypertrophies. Whereas, in conditions of low transport (right), such as Gitelman syndrome or chronic use of thiazides or high plasma K^+^, the DCT segment atrophies. The transport of Na^+^ through NCC is regulated by the WNK4/SPAK cascade. WNK4 activity is modulated by intracellular Cl^–^ levels which are sensitive to plasma K^+^. Low intracellular Cl^–^ activates WNK4, SPAK and NCC, whereas high intracellular Cl^–^ inhibits WNK4, SPAK, and NCC. DCT-mediated Fhht is due to either mutations in WNK4 or mutations in ubiquitin ligase protein complex Cullin-3 and Kelsh-3. In each case, expression of WNK4 is enhanced leading to increased SPAK and NCC phosphorylation.

Similar data were obtained by manipulating WNK4, a kinase that binds (Piechotta et al., 2003), phosphorylates and activates SPAK (Vitari et al., 2005). Studies have shown that mice over-expressing a WNK responsible for the activation of NCC, also demonstrate larger NCC-stained tubules in the transgenic mouse than the wild-type mouse (Lalioti et al., 2006). Thus, all these studies clearly indicate that the mass of the DCT is highly dynamic, following changes in NCC function. Even though the cotransporter utilizes the energy of the Na^+^ gradient to move Cl^–^ into the cell, it is possible that the movement of the cation may also serve a secondary role as a signal for tissue growth and expansion (Figure 6).

### Na^+^/Mg^2+^ Exchanger (NME)

Magnesium (Mg^2+^) is an essential element and the second most abundant intracellular divalent cation in biological systems (Blaine et al., 2015). Mg^2+^ is a key component of many enzymes, present in every cell type in every organism, and therefore critical in essentially every metabolic pathway (Romani, 2013). Adenosine triphosphate (ATP) must bind magnesium to be biologically active, and as such, Mg^2+^ has a significant role in the stability of all polyphosphate compounds (e.g., RNA and DNA) in cells (Allen, 2013). The divalent cation is also critical for the function of protein kinases, including receptor tyrosine kinases (e.g., EFGR, VEGFR, PDGFR, and FGFR), non-receptor tyrosine kinases such as Src, and serine/threonine protein kinases, all of which propagate metabolic signals within cells (Cowan, 2002; Zou et al., 2019). Although abundant and readily bioavailable, Mg^2+^ cannot cross biological membranes, so transport proteins must facilitate Mg^2+^ movement into and out of cells and intracellular compartments (Romani and Maguire, 2002).

Transcripts of the human Na^+^/Mg^2+^ exchangers SLC41A1 (1q31-32), SLC41A2 (12q23.3), and SLC41A3 (3q21.2) mediates the electrogenic transport of 1 Na^+^ ion and 1 Mg^2+^ ion across the plasma membrane (Wabakken et al., 2003; Sahni et al., 2007; Kolisek et al., 2008; Quamme, 2010). cAMP signaling is an important second messenger that regulates processes such as neurotransmitter synthesis (Guseva et al., 2014), ganglion synaptic transmission (Patterson et al., 1996), inflammatory response (Park et al., 2008; Chang et al., 2013), myocardial atrophy (Brown et al., 2012), and transcription factor regulation (Metz and Ziff, 1991). Hormones like glucagon (liver) and adrenaline (muscle) are two examples of extracellular first messengers that bind to G-protein coupled receptors in the plasma membrane and signal transmembrane adenylyl cyclases, anchored to the inner leaflet of the plasma membrane, to convert ATP into cAMP (Rahman et al., 2013). Stimulation of cAMP-dependent protein kinase A (PKA) induces phosphorylation of multiple proteins to evoke specific cellular reactions (Brown et al., 2012). Several functional studies have linked cAMP-activated PKA and PKC to the phosphorylation mediated regulation of the Na^+^/Mg^2+^ exchanger isoform 1 (NME1) (Wabakken et al., 2003; Kolisek et al., 2008; Quamme, 2010; Kolisek et al., 2012).

In the DCT of the kidney, transcellular Mg^2+^ reabsorption is mediated by an apical cation channel (TRPM6) and a basolateral sodium-dependent exchanger (NME1). The expression of an apical K^+^ channel establishes a favorable luminal potential for Mg^2+^ absorption across the apical cytoplasmic membrane (Blaine et al., 2015). Basolateral Na^+^/K^+^ ATPase activity provides the driving force for NME1 extrusion of Mg^2+^ (Goytain and Quamme, 2005; Kolisek et al., 2008). Hypomagnesemia (acute Mg^2+^ deficiency) has been associated with diabetes, anxiety disorders, migraines, osteoporosis, cardiovascular disease, hypertension, and stroke (Larsson et al., 2008; Romani, 2013). While Mg^2+^ is clearly essential to human health, it could more accurately described as a physiological cofactor rather than a signaling factor. As a result, Na^+^ movement through NMEs doesn’t necessarily represent a signaling cascade as much as a driving force for Mg^2+^ homeostasis.

## Cell Cycle, Growth, and Proliferation

The interphase component of the pluripotent eukaryotic cell cycle is subdivided into a growth phase (G1), amassing of energy and DNA replication phase (S), and division preparation phase (G2) (see Figure 5). During the mitotic phase (M) of the cell cycle, the parent cell’s chromosomes are first divided equally between the sister cells (mitosis) and then the two distinct daughter cells are formed by the division of the parent cell’s cytoplasm (cytokinesis) (Kapinas et al., 2013). In 1960, Stubblefield and Mueller demonstrated that increases in extracellular NaCl concentrations from 120 to 220 mM shifted HeLa cells in culture from rapid proliferation (hyperplasia) to increased size (hypertrophy). Interestingly, despite increased cellular size, the DNA per cell remained constant against various extracellular NaCl concentrations, suggesting that high-salt treatment blocks the cell cycle between G1 and S phase (Stubblefield and Mueller, 1960). In the early 1970s, it was shown that mitosis is activated by changes in the electrical membrane potential and increases in the intracellular Na^+^ concentration (Cone and Tongier, 1971, 1973). For instance, DNA synthesis was induced in spinal cord neurons isolated from chick embryos by exposure to ouabain, veratridine, or the ionophore gramicidin (Cone and Cone, 1976). Increased intracellular Na^+^ (1.3 to 2-fold) was also demonstrated in CHO cells during the late S phase and mitosis (Marakhova et al., 1987). As far back as 1926, Dr. Charles Packard observed that the [Na^+^] in the blood of tumor-bearing rats was 25% higher than normal when the tumor was actively growing and 60% higher still when the tumor was receding (Packard, 1926). Amiloride, a compound that inhibits sodium influx and proliferation of normal cells (Koch and Leffert, 1979; Villereal, 1981) reduced tumor growth, tumor cell proliferation, and intra-nuclear Na^+^ content (Sparks et al., 1983).

## Cell Motility

Every cell in the body requires at some point in its life cycle the capacity to move either prior to terminal differentiation or to maintain tissue homeostasis (Schwab et al., 2012). Angiogenesis (Stupack and Cheresh, 2004), wound healing (Martin and Leibovich, 2005; Blikslager et al., 2007; Dignass, 2001), immune response (Friedl and Weigelin, 2008; Nourshargh et al., 2010; Silva, 2010), gastro-intestinal barrier preservation (Yen and Wright, 2006), and neuronal development (Komuro and Rakic, 1998; Komuro and Kumada, 2005; Valiente and Marín, 2010) are all cellular processes dependent on cell motility. Cellular migration machinery (Mogilner and Oster, 2003; Insall and Machesky, 2009; Cramer, 2010), cell adhesion receptors (Jones et al., 2006), and chemokine receptors (Mañes et al., 1999), are just some of the components that need to be asymmetrically distributed to the front and rear poles of the cell for persistent cell migration. Several ionic mechanisms also have significant roles in cell motility. Ca^2+^-sensitive calpain proteases contribute to disassembly of focal adhesion components at the rear pole of migrating cells (Chan et al., 2010). Integrin adhesion has also been shown to be regulated by the activity of various K^+^ channels (Artym and Petty, 2002; Arcangeli and Becchetti, 2006). Coordinated attachment and release of the focal adhesion contacts between cells and the extracellular matrix involves integrins and the Na^+^/H^+^ exchanger (NHE1) (Plopper et al., 1995; Hynes, 2002). Polarization and directed movement of migrating cells requires both cytoskeletal anchoring and ion transport activity of NHE1. Wound healing assays demonstrated that mutations impairing either function resulted in slower fibroblast cell migration than wild type cells (Denker and Barber, 2002). Regulation of intracellular pH and efficient chemotaxis are dependent on NHE1 expression to the leading edge of polarized cells. Adhesion of these migrating cells to the extracellular matrix is also dependent on the extracellular pH (Lehenkari and Horton, 1999; Eble and Tuckwell, 2003; Patel and Barber, 2005; Krähling et al., 2009; Paradise et al., 2011). Taken together, these studies suggest that Na^+^ likely has an indirect role in cell migration as the activity of NHE1 more likely contributes to the extracellular pH microenvironment.

## Cell Metabolism

In order to overcome the Donnan forces associated with macromolecules and organic metabolites unable to diffuse across the plasma membrane, mammalian cells transport Na^+^ (and osmotically obligatory water) out of the cell primarily by the action of the Na^+^/K^+^ ATPase (Chatton et al., 2016). The energy produced by the 10-fold Na^+^ gradient created by the hydrolysis of ATP is utilized by astrocytes to clear neurotransmitters and recycle metabolic waste products (Weber and Barros, 2015). Another study has demonstrated that increased Na^+^ uptake in adipose tissue promotes increased plasma adiponectin via stimulation of peroxisome proliferator-activated receptors. Enhanced adiponectin, in turn, inhibits renal SGLT2 function and ultimately proximal tubule reabsorption of Na^+^ and glucose. Interestingly, this metabolic pathway is impaired by diabetes and thereby results in hyperglycemia-induced Na^+^ retention (Zhao et al., 2016). Chronic hyponatremia, a common disorder in elderly people, has been linked to increased skeletal fractures (Gankam Kengne et al., 2008; Sandhu et al., 2009). *In vitro* experiments demonstrated that decreased extracellular Na^+^ in the medium of cultured bone marrow monocytes dose-dependently increased osteoclastogenesis and osteoclastic resorptive activity. One possible mechanism through which extracellular [Na^+^] might inversely regulate this resorptive activity is through the Na^+^-dependent vitamin C transporter (Barsony et al., 2011). It is well documented that vitamin C protects osteoblasts (Wilson and Dixon, 1989) and osteoclasts (Xiao et al., 2005) from reactive oxygen species accumulation and oxidative stress, and that decreased extracellular [Na^+^] inhibits vitamin C transporter activity. Indeed, bone mineral density studies in humans have inversely associated the oxidative stress response as a mechanism for chronic hyponatremia-induced pathologies (Basu et al., 2001).

## Conclusion

The extracellular to intracellular Na^+^ gradient maintained by the action of the Na^+^/K^+^ ATPase provides an electrochemical driving force for multiple Na^+^-dependent transport mechanisms. Overall, it is clear that President *John F. Kennedy* was correct in stating that “*we are all tied to the sea*” and that Na^+^ is critical to all aspects of human physiology. In this review we have endeavored to both highlight the functional role of these transporters and exchangers in human health and disease and consider their potential role as components of intracellular signaling pathways using Na^+^ ions.

## Author Contributions

KG and ED have contributed to the writing, editing, creation of Figures, and have approved the final version of the manuscript.

## Conflict of Interest

The authors declare that the research was conducted in the absence of any commercial or financial relationships that could be construed as a potential conflict of interest.
